# Endoloop-assisted endoscopic removal of over-the-scope and full-thickness resection device clips: Prospective study

**DOI:** 10.1055/a-2784-8740

**Published:** 2026-03-06

**Authors:** Tobias Blasberg, Lukas Hiebel, Moritz Meiborg, Johannes Richl, Florian Alexander Michael, Ali Seif Amir Hosseini, Ahmad Amanzada, Volker Ellenrieder, Juergen Hochberger, Wedi Edris

**Affiliations:** 1 9206 Gastroenterology, Gastrointestinal Oncology and Interventional Endoscopy, Sana Klinikum Offenbach GmbH, Offenbach, Germany; 2 14903 Department of Hepatology and Gastroenterology, Charité – Universitätsmedizin Berlin Campus Charite Mitte, Berlin, Germany; 3 27177 Clinic for Gastroenterology, Gastrointestinal Oncology and Endocrinology, University Medical Center Göttingen, Göttingen, Germany; 4 243538 Department of Medicine I Gastroenterology Hepatology Pneumology Allergology Endocrinology Diabetology, Medical Clinic 1, University Hospital Frankfurt, Frankfurt, Germany; 5 27177 Department of Clinical and Interventional Radiology, University Medical Center Göttingen, Göttingen, Germany

**Keywords:** Endoscopy Upper GI Tract, Non-variceal bleeding, Ulcers (peptic and other), Precancerous conditions & cancerous lesions (displasia and cancer) stomach, Endoscopy Lower GI Tract, Polyps / adenomas / ..., Lower GI bleeding

## Abstract

**Background and study aims:**

The over-the-scope (OTS) clip system is an established tool for endoscopic management of gastrointestinal bleeding, perforations, fistulas, and, in its modified form as the full-thickness resection device (FTRD), for resection of gastrointestinal lesions. In selected cases, clip removal is required. Conventional techniques often demand costly devices and may be time-consuming. This study evaluated a novel, simple technique for endoscopic removal of OTS and FTRD clips using an endoloop, focusing on feasibility and safety.

**Patients and methods:**

This prospective single-center study included patients who had undergone OTS or FTRD clip placement and required clip removal between March 2021 and October 2024. The removal procedure involved positioning an endoloop underneath the clip. Follow-up endoscopy within 6 months assessed clip detachment. The primary endpoint was the success rate for clip removal; the secondary endpoint was incidence of adverse events (AEs).

**Results:**

A total of 18 patients underwent endoloop-assisted removal of OTS (50%, 9/18) or FTRD (50%, 9/18) clips. Endoloop placement was performed at a median of 94 days (range: 27–472) after OTS clip placement and 97 days (range: 0–274) after EFTR. Follow-up endoscopy confirmed clip detachment after a median of 107 days (range: 33–220) for OTS and 114 days (range: 5–203) for FTRD clips. Clip removal was successful in all patients (100%), with no AEs observed.

**Conclusions:**

Endoloop-assisted removal of OTS and FTRD clips appears to be effective and safe, representing an addition to current options for endoscopic clip removal.

## Introduction

The over-the-scope (OTS) clip (Ovesco Endoscopy AG, Tuebingen, Germany) system is an established endoscopic device used for management of gastrointestinal bleeding, perforations, and fistulas 1 2 3 . A derivative of this system, the full-thickness resection device (FTRD; Ovesco Endoscopy AG, Tuebingen, Germany) has proven effective for endoscopic full-thickness resection (EFTR) of gastrointestinal lesions 4 5 . Although both OTS clips and FTRD clips are designed and approved for long-term implantation, their removal may be required in certain clinical scenarios, such as clip misplacement, luminal obstruction, incomplete resection following EFTR, or at patient request. Several techniques for clip removal have been described, including the remOVE system (Ovesco Endoscopy AG, Tuebingen, Germany), grasping forceps, Nd:YAG laser, argon plasma coagulation (APC), endoscopic mucosal resection/endoscopic submucosal dissection (EMR/ESD), and use of ice-cold saline 6 . Among these, the remOVE system is the most extensively studied and has demonstrated high efficacy and safety. A systematic review reported high success rates for OTS and FTRD clip removal, with success rates of 90.3% and 94.5%, respectively 6 . Despite these favorable outcomes, its clinical adoption remains limited, potentially due to restricted availability and cost-effectiveness. Furthermore, evidence on alternative removal techniques is sparse and largely limited to case reports, with many methods unsuitable for clips deeply embedded in the gastrointestinal wall.

The endoloop (detachable snare) is a widely used endoscopic device, primarily employed for ligation of large pedunculated polyps to prevent bleeding 7 8 9 . Beyond this application, the endoloop has demonstrated utility in managing colonic diverticular hemorrhage and in closing ESD-induced mucosal defects 10 11 12 . Jung et al. described an innovative use of an endoloop for hemostasis in a duodenal ulcer that had re-bled beneath the base of a previously applied traumatic OTS clip 13 . During follow-up, both the OTS clip and the endoloop spontaneously detached. This observation provided the rationale for investigating endoloop-assisted removal of OTS and FTRD clips.

The present study prospectively evaluated feasibility and safety of endoloop-assisted removal of OTS and FTRD clips in a clinical setting.

## Patients and methods

### Study design and patient selection

This prospective study was conducted at our endoscopy center between March 2021 and October 2024. Patients aged ≥ 18 years who had previously undergone OTS or FTRD clip placement in the upper or lower gastrointestinal tract and in whom clip removal was indicated were eligible for inclusion. Indications for clip removal included the potential need for re-intervention following EFTR or endoscopic resection, patient preference, luminal obstruction caused by the clip, and misplacement of the clip. Endoloop-assisted removal was predefined as the exclusive clip-removal technique within the study protocol. This study was conducted at Sana Klinikum Offenbach, Germany. All endoloop placements were performed by five experienced endoscopists. Informed consent was obtained from all participants. The study protocol was approved by the institutional review board (2025–3986-evBO) and was conducted according to the Declaration of Helsinki.

### Endoloop technique

**Fig. 1:**
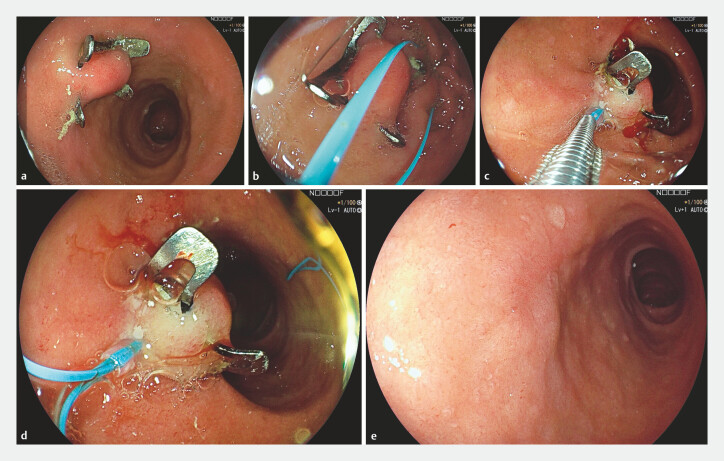
**a, b, c, d, e** Endoloop-assisted removal of an FTRD clip: stepwise endoscopic procedure and follow-up.

Endoloop-assisted removal was performed using the PolyLoop Ligation Device (Olympus, Tokyo, Japan). The endoloop was positioned underneath the OTS or FTRD clip, tightened, and then released from the hook. Successful endoloop placement was verified by an immediate color change of the tissue within the OTS or FTRD clip, indicating ischemia caused by the gradual constrictive force of the endoloop. At the discretion of the treating endoscopist, the residual end of the endoloop was removed using a dedicated loop cutter (Olympus, Tokyo, Japan). The stepwise procedure is demonstrated in Fig. 1 . The procedure was performed using a gastroscope (EG-760Z, Fujifilm, Tokyo, Japan) for the upper gastrointestinal tract and a colonoscope (EC-760Z, Fujifilm, Tokyo, Japan) for the lower gastrointestinal tract. All interventions were conducted under propofol sedation with consistent carbon dioxide insufflation. Clinically asymptomatic patients were discharged on the same day following a standardized post-procedural observation period of at least 2 hours, including vital sign monitoring and clinical assessment for signs of perforation. Patients were provided with direct contact information and instructed to report immediately in case of abdominal pain or other symptoms suggestive of complications.

### Endpoints and follow-up

The primary endpoint of this study was the success rate for clip removal, defined as absence of the clip at the site during follow-up endoscopy. Secondary outcomes included the rate of adverse events (AEs) associated with placement of an endoloop. All patients underwent follow-up endoscopy within 6 months after endoloop placement. The exact timing was determined by the treating endoscopist based on clinical context, but all procedures were performed within this defined interval.

### Statistical analysis

Descriptive statistics were calculated using SPSS version 30 (IBM Corp., Armonk, New York, United States). Continuous variables are presented as median and range; categorical variables are reported as frequencies and percentages.

## Results

### Patient and procedure characteristics

A total of 18 patients underwent endoloop-assisted removal of OTS (n = 9) and FTRD (n = 9) clips during the study period from March 2021 to October 2024. Baseline characteristics are presented in Table 1 . Mean age of the study cohort was 66.3 ± 10.4 years, with a trend toward a higher mean age in the OTS clip group (68.6 ± 9.3 years) compared with the FTRD clip group (64.0 ± 11.1 years). The overall sex distribution was balanced (50.0% female), although a higher proportion of females was observed in the OTS clip group (66.7%) compared with the FTRD clip group (33.3%). The most common comorbidities included cardiovascular disease (55.6%), obesity (50.0%), pulmonary disease (33.3%), and diabetes mellitus (33.3%), with no major differences between groups. The majority of clips were located in the upper gastrointestinal tract (66.7%), but their distribution in the upper gastrointestinal tract varied by clip type. FTRD clips were predominantly placed in the duodenum (55.6%), whereas OTS clips were more frequently applied in the stomach (44.4%). In the lower gastrointestinal tract (33.3%), FTRD and OTS clips were most commonly placed in the cecum (22.2% and 11.1%, respectively), followed by the descending colon (both 11.1%) and the rectum (0% and 11.1%, respectively).

**Table:** **Table 1** Baseline characteristics of enrolled patients.

	Total population (N = 18)	OTS clip (n = 9)	FTRD (n = 9)
**Age, years**			
Mean ± SD	66.3 ± 10.4	68.6 ± 9.3	64.0 ± 11.1
Sex, n (%)			
Female	9 (50.0)	6 (66.7)	3 (33.3)
Male	9 (50.0)	3 (33.3)	6 (66.7)
**Comorbidities, n (%)**			
Cardiovascular disease	10 (55.6)	5 (55.6)	5 (55.6)
Renal disease	2 (11.1)	2 (22.2)	0 (0.0)
Pulmonary disease	6 (33.3)	3 (33.3)	3 (33.3)
Diabetes mellitus	6 (33.3)	2 (22.2)	4 (44.4)
Obesity	9 (50.0)	4 (44.4)	5 (55.6)
Tobacco use (past or current)	8 (44.4)	3 (33.3)	5 (55.6)
Alcohol use (past or current)	2 (11.1)	0 (0.0)	2 (22.2)
**Clip location, n (%)**			
Upper gastrointestinal tract	12 (66.7)	6 (66.7)	6 (66.7)
Stomach	5 (27.8)	4 (44.4)	1 (11.1)
Duodendum	7 (38.9)	2 (22.2)	5 (55.6)
Lower gastrointestinal tract	6 (33.3)	3 (33.3)	3 (33.3)
Cecum	3 (16.7)	1 (11.1)	2 (22.2)
C. descendens	2 (11.1)	1 (11.1)	1 (11.1)
Rectum	1 (5.6)	1 (11.1)	0 (0.0)
**OTS clip size and type, n (%)**			
11/3 (a)	–	6 (66.7)	–
12/3 (a)	–	1 (11.1)	–
12/3 (t)	–	1 (11.1)	–
14/3 (a)	–	1 (11.1)	–
**Indication for OTS clip, n (%)**			
Hemostasis	–	3 (33.3)	–
OTS clip type (a)	–	3 (100.0)	–
OTS clip type (t)	–	0 (0.0)	–
Perforation	–	0 (0.0)	–
Secondary prophylaxis	–	6 (66.7)	–
OTS clip type (a)	–	5 (83.3)	–
OTS clip type (t)	–	1 (16.7)	–
**Indication for FTRD, n (%)**			
Recurrent or residual adenoma	–	–	4 (44.4)
Non-lifting adenoma	–	–	2 (22.2)
Suspected adenocarcinoma (T1)	–	–	2 (22.2)
Submucosal lesion	–	–	1 (11.1)
**Indications for clip removal using an endoloop, n (%)**			
Obstruction	0 (0.0)	0 (0.0)	0 (0.0)
Potential reintervention after EFTR or endoscopic resection	10 (55.6)	2 (22.2)	7 (77.8)
Clip removal for misplaced clip	0 (0.0)	0 (0.0)	1 (11.1)
Clip removal due to pain	0 (0.0)	0 (0.0)	0 (0.0)
Clip removal at patient’s request	8 (44.4)	7 (77.8)	1 (11.1)
**Time of clip in situ before endoloop placement, days**			
Median (range)	94 (0–472)	94 (27–472)	97 (0–274)
**Duration of procedure, minutes**			
Total procedure duration, median (range)	23 (15–49)	23 (15–41)	20 (16–49)
Endoloop placement, median (range)	4 (2–11)	3 (2–8)	5 (3–11)
a, atraumatic; FTRD, full-thickness resection device; OTS, over-the-scope; t, traumatic; SD, standard deviation.			

OTS clips were primarily used for secondary prophylaxis (66.7%) to prevent delayed bleeding or perforation after wide-field endoscopic resection, whereas hemostasis accounted for 33.3% of cases. The atraumatic OTS clip type was predominantly used for both prophylaxis (83.3%) and hemostasis (100%). EFTR was performed for recurrent or residual adenoma (44.4%), non-lifting adenoma (22.2%), suspected adenocarcinoma (22.2%), and submucosal lesions (11.1%). Primary indications for clip removal were potential need for reintervention following EFTR or endoscopic resection (OTS clip 22.2% vs. FTRD clip 77.8%), patient request (OTS clip 77.8% vs. FTRD clip 11.1%), and misplaced clips (OTS clip 0.0% vs. FTRD clip 11.1%). Patient-driven requests were mainly related to the perception that the clip was no longer therapeutically required, concerns about long-term implant retention (e.g. foreign-body sensation), and anticipated limitations of future magnetic resonance imaging examinations due to clip-related artifacts. Other potential indications for clip removal, such as clip-associated pain or luminal obstruction, were not observed in our study.

On average, the endoloop was applied 94 days (range: 27–472 days) after OTS clip placement and 97 days (range: 0–274 days) after EFTR. Because the OTS clips in our study were not used for treatment of perforations, clip removal was considered acceptable after a relatively short time interval. In one case, an endoloop was applied as early as 27 days after clip placement, and in three additional cases, between 35 and 42 days. In contrast, all other OTS clips remained in situ for 94 to 472 days before endoloop application. Following successful EFTR, endoloop placement was generally delayed to ensure sufficient tissue healing. In all but one case, the interval between EFTR and endoloop placement ranged from 71 to 274 days. The single exception was due to clip misdeployment: During EFTR, the FTRD clip was released, and at the moment of deployment, the target lesion partially slipped out of the clip, resulting in incomplete capture and failure to achieve a full-thickness resection. To facilitate subsequent detachment of the clip and allow for accurate reassessment of the resection site, an endoloop was placed around the FTRD clip during the same procedure.

Median total procedure duration was 23 minutes (range: 15–49 minutes), with similar durations in the OTS clip (23 minutes; range: 15–41) and FTRD subgroups (20 minutes; range: 16–49). Total procedure duration was defined as time from endoscope insertion to final withdrawal. Median time required for endoloop placement was 4 minutes (range: 2–11 minutes), with subgroup medians of 3 minutes (range: 2–8) in the OTS clip group and 5 minutes (range: 3–11) in the FTRD group. Endoloop placement time was defined as the interval from insertion of the endoloop through the endoscope to its release from the hook. In 11 of 18 procedures (61.1%), the residual end of the endoloop was removed using a loop cutter.

### Outcomes

Endoloop-assisted clip removal was successfully performed in all 18 patients, achieving a 100% success rate for both OTS and FTRD clips. No AEs related to the procedure, including bleeding, perforation, or other complications, were observed in any patient. Endoscopic follow-up was performed in all patients (100%). Median interval between endoloop application and endoscopic follow up to confirm clip detachment was performed 107 days (range: 33–220) after OTS clip placement and 114 days (range: 5–203) after EFTR. Detailed outcome data are summarized in Table 2 .

**Table:** **Table 2** Outcome analysis of endoloop-assisted clip removal.

	Total population (N = 18)	OTS clip (n = 9)	FTRD (n = 9)
**Clip removal success rate, n (%)**	18 (100.0)	9 (100.0)	9 (100.0)
**Follow-up, n (%)**	18 (100.0)	9 (100.0)	9 (100.0)
**Adverse events due to endoloop placement, n (%)**	0 (0.0)	0 (0.0)	0 (0.0)
Bleeding	0 (0.0)	0 (0.0)	0 (0.0)
Perforation	0 (0.0)	0 (0.0)	0 (0.0)
Respiratory	0 (0.0)	0 (0.0)	0 (0.0)
Others	0 (0.0)	0 (0.0)	0 (0.0)
**Interval between endoloop placement and follow-up endoscopy, days**			
Median (range)	110 (5–220)	107 (33–220)	114 (5–203)
Mortality during follow-up, n (%)	0 (0.0)	0 (0.0)	0 (0.0)
FTRD, full-thickness resection device; OTS, over-the-scope.			

## Discussion

This prospective study introduces a novel technique for endoscopic removal of OTS and FTRD clips by using an endoloop. The primary endpoint of this study, defined as endoscopically evaluated successful clip removal, was reached in all cases. Noteworthy, no AEs occurred during the study period, underscoring the favorable safety profile of this new technique. To our knowledge, this is the first study investigating feasibility, safety, and efficacy of endoloop-assisted removal of OTS and FTRD clips.

Although OTS and FTRD clips are approved for long-term implantation, spontaneous detachment is commonly observed over time. However, timing of detachment remains unpredictable and depends on several factors, including clip type, amount and composition of compressed tissue, and individual tissue response to mechanical compression. Hu et al. reported a spontaneous OTS clip detachment rate of 27.8% over a mean follow-up of 30.4 ± 9.3 months in patients treated for iatrogenic perforations 14 . Detachment rates for FTRD clips have been reported to range between 68.8% and 84.4% at 3-month follow-up after EFTR 15 16 . Active clip removal may become necessary in specific clinical situations, including incomplete resection requiring further endoscopic therapy or surveillance, clip misplacement, luminal obstruction, or patient request. Notably, with an R0 resection rate of approximately 81.8% following EFTR 17 , further intervention may be required in up to one-fifth of cases, emphasizing the relevance of a safe and accessible removal strategy.

Several techniques for OTS and FTRD clip removal have been described, including the remOVE system, grasping forceps, EMR/ESD, application of cold saline, Nd:YAG laser, and APC. Among these, the remOVE system is supported by the most comprehensive data. A systematic review by Ou et al. summarized 14 case reports and five clinical trials investigating various clip removal methods, encompassing a total of 167 patients (23 from case reports and 144 from clinical trials) 6 . All studies were retrospective, single-arm observational in design. Alternative methods to the remOVE system were described only in case reports and one single clinical trial, comprising 19 patients in total 18 19 20 21 22 23 24 . These studies reported high technical success rates (100%) with minimal AEs. In contrast, the remOVE system was used in all remaining patients across the included studies 15 25 26 27 28 29 30 31 . The clinical trials investigating the remOVE system reported pooled success rates of 90.3% for OTS clip and 94.5% for FTRD clip removal 6 . Despite these favorable outcomes, the system has not seen widespread clinical adoption. Contributing factors may include its limited availability and associated moderate to high costs, which reduce its overall cost-effectiveness despite demonstrated efficacy. Data on alternative removal methods remain scarce and are largely limited to case reports. Moreover, many of these methods are unsuitable for clips that are deeply embedded in the mucosa or submucosa. In this context, our findings demonstrate that endoloop-assisted clip removal achieves a comparable success rate (100.0%) and may represent a low-cost, widely accessible, and clinically viable alternative. Table 3 summarizes key differences between endoloop-assisted removal and the remOVE system.

**Table:** **Table 3** Comparison of endoloop-assisted removal versus the remOVE System for OTS and FTRD clip extraction.

	Endoloop-assisted removal	remOVE System
**Device requirements**	Endoloop (e.g., PolyLoop Ligation Device) ^*^	remOVE DC impulse and DC Cutter Set (DC cutter, SecureCap, Grasper, Shield) ^†^
**Procedure duration**	Short ^‡^	Specific device-related duration not reported ^§^
**Confirmation of clip removal**	Requires follow-up endoscopy; detachment is delayed and cannot be confirmed immediately	Immediate confirmation during procedure
**Clip removal success rate, %**	100.0 (present study)	90.3–94.5 6
**Adverse events**	None observed (present study)	Minor adverse events (mucosal tears, minor bleeding, thermal injury) 23 24 25
**Cost**	Low ^¶^	Moderate ^††^
^*^ Olympus, Tokyo, Japan. ^†^ Ovesco Endoscopy AG, Tuebingen, Germany. ^‡^ Median duration of endoloop placement: 4 minutes; data derived from the present study. ^§^ Previous studies on the remOVE system reported only total procedure duration (mean: 47–54 minutes) 25 26 , without specifying duration needed for clip removal. ^¶^ Estimated device cost in Germany < €50. ^††^ Estimated device cost for the remOVE DC Cutter Set in Germany > €350; remOVE DC Impulse device can be borrowed from the manufacturer or purchased. FTRD, full-thickness resection device; OTS, over-the-scope.		

No AEs, including intraprocedural or delayed bleeding and perforation, were observed in our study. The endoloop-assisted removal technique may inherently reduce risk of bleeding via gradual, mechanical strangulation at the clip base. This principle of progressive tissue compression has demonstrated safety in other endoscopic contexts, such as treatment of colonic diverticular bleeding and prophylactic ligation of large pedunculated polyps. For instance, Kobayashi et al. reported that detachable endoloop ligation for colonic diverticular hemorrhage was both safe and effective, with no delayed perforation observed 10 . Similarly, Ji et al. demonstrated successful endoloop ligation of large pedunculated polyps without any associated AEs 9 . In addition, because the clip detaches progressively and remains structurally intact, risk of perforation due to sharp edges from partially dislodged clips may be minimized. To date, major AEs related to OTS or FTRD clip removal using the remOVE system or other techniques have not been reported 6 . However, minor events such as superficial thermal injury, mucosal tears, and minor bleeding have been documented. Schmidt et al. reported superficial thermal damage in 100% of cases using the remOVE system 26 . Mucosal tears occurred in 9.1% and 1.4% of cases in the studies by Schmidt et al. and Caputo et al., respectively 26 27 . Minor bleeding was observed in three clinical trials, with a pooled rate of 7% 25 26 27 . Therefore, the endoloop-assisted method may represent a safe and potentially advantageous alternative for the removal of OTS and FTRD clips.

In our study, median time required for endoloop placement was 4 minutes (range: 2–11 minutes), with comparable durations observed in both the OTS and FTRD subgroups. Comparative data on the exact duration of clip removal using alternative techniques are limited. To date, only two clinical studies have reported total procedure times for clip removal using the remOVE system 25 26 . In the retrospective study by Bauder et al., mean total procedure times were 54 minutes for FTRD and 49 minutes for OTS clip removal; however, no separate data were provided for the clip removal step itself, precluding a direct comparison with the endoloop-assisted technique 25 . Similarly, Schmidt et al. reported a mean procedure time of 47 minutes (range, 35–75 minutes), without specifying whether this duration referred solely to clip removal or the entire endoscopic procedure 26 . Given the lack of procedure-specific time measurements in existing studies, a prospective comparative trial is needed to adequately assess procedure efficiency. Nonetheless, the findings from our study suggest that endoloop-assisted clip removal may represent a time-efficient alternative to currently established methods.

Currently, no guidelines exist defining optimal timing for removal of retained OTS or FTRD clips. In the present study, endoloop placement was mainly performed at routine follow-up endoscopy, typically 3 to 6 months after complex endoscopic resection or EFTR, reflecting standard clinical practice rather than a predefined optimal interval. Notably, our data demonstrate that endoloop-assisted removal is technically feasible across a wide temporal range, with successful FTRD clip removal up to 274 days and OTS clip removal up to 472 days after initial deployment. Given that all clips were successfully removed and the cohort size was limited, no optimal timing for endoloop-assisted clip removal can be defined based on our results. Instead, the key prerequisite is sufficient tissue healing to reduce risk of bleeding or perforation after clip removal, which primarily depends on depth of clip anchoring. OTS clips placed for secondary prophylaxis after endoscopic resection and FTRD clips following EFTR are typically anchored deeply within the gastrointestinal wall, whereas OTS clips used solely for hemostasis are usually more superficially embedded. Accordingly, in settings involving deep tissue anchoring, a longer healing interval appears advisable. Once adequate tissue healing is ensured, endoloop-assisted clip removal can be safely performed, most commonly during scheduled follow-up endoscopy.

Data on the interval between endoloop application and actual clip detachment are currently lacking. In our cohort, follow-up endoscopy was scheduled within 6 months and performed at a median of 110 days after endoloop placement, which did not allow precise determination of the exact time point of detachment in most patients. The earliest endoscopic confirmation of OTS clip detachment was obtained 33 days after endoloop placement. An earlier confirmation at 5 days was observed for an FTRD clip; however, this case followed clip misdeployment without full-thickness resection and, therefore, has limited interpretability. The second earliest confirmation of clip detachment after successful EFTR was observed at 42 days. Overall, detachment kinetics appear variable and are likely influenced by clip type as well as the extent of tissue ingrowth and fixation. Prospective studies with predefined short-interval follow-up are warranted to better characterize time to detachment and to inform individualized planning of follow-up and potential re-intervention.

The underlying mechanism facilitating clip detachment after endoloop ligation is likely multifactorial. The endoloop exerts a gradual constrictive force around the clip base and the associated tissue, resulting in localized ischemia that induces mucosal inflammation and necrosis of the compressed tissue. This ischemia-induced necrosis weakens clip adherence to the tissue, ultimately leading to detachment. In addition, mechanical factors such as repetitive peristaltic movements and external forces may contribute to mechanical fatigue and progressive loosening of the clip. Further elucidation of these biological and mechanical processes through histopathological and biomechanical studies will be essential to optimize the technique and ensure reproducibility.

Despite the promising safety and efficacy profile, endoloop-assisted clip removal requires specific technical considerations. Due to the soft and flexible nature of the endoloop, precise placement can be challenging, particularly in anatomically difficult locations such as the duodenal flexure or colonic flexures. Nonetheless, in our study, successful application was achieved in all cases, including those involving flexural regions. Successful deployment requires sufficient expertise in endoloop use, as well as effective coordination between the endoscopist and the assisting staff. A critical step for successful removal is complete encirclement of all clip brackets to ensure adequate ligation. Importantly, timing of clip detachment following endoloop application is unpredictable and primarily depends on the extent of tissue ingrowth and depth of clip embedding within the gastrointestinal wall.

This study has several limitations. It is a single-center study with a relatively small sample size, reflecting the infrequent clinical indication for active clip removal. The non-comparative study design precludes direct evaluation of endoloop-assisted removal against established methods such as the remOVE system. In addition, lack of a control group (e.g., no active removal) limits conclusions regarding the natural course of clip retention versus active removal. Furthermore, OTS clips placed for treatment of acute gastrointestinal perforation were not included in this study. This was not due to an exclusion criterion; rather, during the study period, there were no patients treated with an OTS clip for acute perforation who subsequently required clip removal. Consequently, no definitive conclusions can be drawn regarding feasibility or safety of endoloop-assisted removal in this specific clinical setting. A randomized controlled trial comparing endoloop-assisted clip removal with the remOVE system is warranted to elucidate the relative clinical benefits, safety profile, and procedural efficiency of this novel technique.

## Conclusions

This study is the first to evaluate endoloop-assisted removal of OTS and FTRD clips. The technique is simple, effective, widely available, and demonstrated a high success rate with no observed AEs.

## References

[1] Kirschniak A Kratt T Stuker D A new endoscopic over-the-scope clip system for treatment of lesions and bleeding in the GI tract: first clinical experiences Gastrointest Endosc 2007 66 162 167 10.1016/j.gie.2007.01.034 17591492

[2] Wedi E Gonzalez S Menke D One hundred and one over-the-scope-clip applications for severe gastrointestinal bleeding, leaks and fistulas World J Gastroenterol 2016 22 1844 1853 10.3748/wjg.v22.i5.1844 26855543 PMC4724615

[3] Kobara H Mori H Nishiyama N Over-the-scope clip system: A review of 1517 cases over 9 years J Gastroenterol Hepatol 2019 34 22 30 10.1111/jgh.14402 30069935

[4] Ferlitsch M Hassan C Bisschops R Colorectal polypectomy and endoscopic mucosal resection: European Society of Gastrointestinal Endoscopy (ESGE) Guideline - Update 2024 Endoscopy 2024 56 516 545 10.1055/a-2304-3219 38670139

[5] Schmidt A Bauerfeind P Gubler C Endoscopic full-thickness resection in the colorectum with a novel over-the-scope device: first experience Endoscopy 2015 47 719 725 25763833 10.1055/s-0034-1391781

[6] Ou YH Kong WF Li LF Methods for endoscopic removal of over-the-scope clip: a systematic review Can J Gastroenterol Hepatol 2020 2020 5716981 10.1155/2020/5716981 PMC7468599 32908852

[7] Hachisu T A new detachable snare for hemostasis in the removal of large polyps or other elevated lesions Surg Endosc 1991 70 74 10.1007/BF00316840 1948617

[8] Iishi H TM Narahara H Iseki K Endoscopic resection of large pedunculated colorectal polyps using a detachable snare Gastrointest Endosc 1996 44 594 597 10.1016/s0016-5107(96)70015-9 8934168

[9] Ji J-S Lee S-W Kim T Comparison of prophylactic clip and endoloop application for the prevention of postpolypectomy bleeding in pedunculated colonic polyps: a prospective, randomized, multicenter study Endoscopy 2014 46 598 604 10.1055/s-0034-1365515 24830400

[10] Kobayashi K Miura N Furumoto Y Clinical outcomes of endoscopic detachable snare ligation for colonic diverticular hemorrhage: Multicenter cohort study Digest Endosc 2024 36 1357 1366 10.1111/den.14874 38965645

[11] Abe S Oda I Mori G Complete endoscopic closure of a large gastric defect with endoloop and endoclips after complex endoscopic submucosal dissection Endoscopy 2015 47 E374 E375 26273768 10.1055/s-0034-1392594

[12] Akutsu D Narasaka T Kobayashi K Newly developed endoscopic detachable snare ligation therapy for colonic diverticular hemorrhage: a multicenter phase II trial (with videos) Gastrointest Endosc 2018 88 370 377 29679691 10.1016/j.gie.2018.04.2337

[13] Jung C Sportes A Ellenrieder V Endoloop rescue therapy for a duodenal ulcer that re-bled under the clip base after application of an 11/3 traumatic over-the-scope clip Endoscopy 2020 52 E253 E254 31995824 10.1055/a-1089-7580

[14] Hu J Yang Y Ge N Long-term assessment of over-the-scope clip (OTSC) behavior after gastric application Minim Invasive Ther Allied Technol 2020 29 86 89 10.1080/13645706.2019.1590417 31144550

[15] Schmidt A Beyna T Schumacher B Colonoscopic full-thickness resection using an over-the-scope device: a prospective multicentre study in various indications Gut 2018 67 1280 1289 28798042 10.1136/gutjnl-2016-313677

[16] Gibiino G Binda C Papparella LG Technical failure during colorectal endoscopic full-thickness resection: the "through thick and thin" study Endoscopy 2024 56 831 839 38754466 10.1055/a-2328-4753

[17] Nabi Z Samanta J Dhar J Device-assisted endoscopic full-thickness resection in colorectum: Systematic review and meta-analysis Dig Endosc 2024 36 116 128 10.1111/den.14631 37422920

[18] von Renteln D Denzer UW Schachschal G Endoscopic closure of GI fistulae by using an over-the-scope clip (with videos) Gastrointest Endosc 2010 72 1289 1296 20951989 10.1016/j.gie.2010.07.033

[19] Fahndrich M Sandmann M Heike M Removal of over the scope clips (OTSC) with an Nd:YAG Laser Z Gastroenterol 2011 49 579 583 10.1055/s-0029-1245871 21557167

[20] Kapadia S Nagula S Kumta NA Argon plasma coagulation for successful fragmentation and removal of an over-the-scope clip Dig Endosc 2017 29 820 821 10.1111/den.12925 28741735

[21] Sedarat A Ginsberg GG Ahmad N An over-the-scope clipping device inadvertently sealing the pylorus: first description of a removal method Gastrointest Endosc 2014 79 711 10.1016/j.gie.2013.11.010 24360653

[22] Mumtaz S Appannagari A Gupta N Two endoscopic resection methods for the removal of an over-the-scope clip Gastrointest Endosc 2015 82 744 10.1016/j.gie.2015.05.029 26099931

[23] Rocha M Kuttner Magalhaes R Maia L Endoscopic removal of two esophageal over-the-scope clips with cold saline solution technique GE Port J Gastroenterol 2018 26 73 74 30675508 10.1159/000487152 PMC6341318

[24] Mudumbi S Velazquez-Avina J Neumann H Anchoring of self-expanding metal stents using the over-the-scope clip, and a technique for subsequent removal Endoscopy 2014 46 1106 1109 10.1055/s-0034-1377916 25268306

[25] Bauder M Meier B Caca K Endoscopic removal of over-the-scope clips: Clinical experience with a bipolar cutting device United Europ Gastroenterol J 2017 5 479 484 10.1177/2050640616671846 PMC5446147 28588877

[26] Schmidt A Riecken B Damm M Endoscopic removal of over-the-scope clips using a novel cutting device: A retrospective case series Endoscopy 2014 46 762 766 10.1055/s-0034-1365493 24770968

[27] Caputo A Schmidt A Caca K Efficacy and safety of the remOVE System for OTSC and FTRD clip removal: data from a PMCF analysis Minimally Invasive Therapy and Allied Technologies 2018 27 138 142 28608741 10.1080/13645706.2017.1335643

[28] Schiffmann L Roth M Kuehn F PEG closure in the second attempt Endosc Int Open 2016 4 E759 760 10.1055/s-0042-107071 27556092 PMC4993881

[29] Andrisani G Soriani P Manno M Colo-rectal endoscopic full-thickness resection (EFTR) with the over-the-scope device (FTRD): A multicenter Italian experience Dig Liver Dis 2019 51 375 381 30377063 10.1016/j.dld.2018.09.030

[30] Meier B Schmidt A Glaser N Endoscopic full-thickness resection of gastric subepithelial tumors with the gFTRD-system: a prospective pilot study (RESET trial) Surg Endosc 2020 34 853 860 31187233 10.1007/s00464-019-06839-2

[31] Valli PV Mertens J Bauerfeind P Safe and successful resection of difficult GI lesions using a novel single-step full-thickness resection device (FTRD) Surg Endosc 2018 32 289 299 10.1007/s00464-017-5676-9 28664442

